# Evaluation of an Emergency Department Lean Process Improvement Program to reduce length of stay

**DOI:** 10.1186/1472-6963-14-S2-P110

**Published:** 2014-07-07

**Authors:** Marian Vermeulen, Therese Stukel, Astrid Guttmann, Brian Rowe, Merrick Zwarenstein, Brian Golden, Amit Nigam, Geoff Anderson, Robert Bell, Michael Schull

**Affiliations:** 1 Institute for Clinical Evaluative Sciences, Toronto, Canada; 2 Institute for Health Policy, Management and Evaluation, University of Toronto, Toronto, Canada; 3 Department of Emergency Medicine, University of Alberta, Edmonton, Canada; 4 Centre for Studies in Family Medicine, Schulich School of Medicine and Dentistry, Western University, London, Canada; 5 Rotman School of Management, University of Toronto, Toronto, Canada; 6 Cass Business School, City University, London, UK; 7 University Health Network, Toronto, Canada

## Background

In recent years, Lean principles have been applied to improve wait times in the Emergency Department (ED). In 2009, an ED Process Improvement Program based on Lean methods was introduced in Ontario as part of a broad strategy to reduce ED length of stay (LOS) and improve patient flow. This study sought to determine the effect of this program on ED wait times and quality of care.

## Methods

We conducted a retrospective cohort study of all ED visits at program and control sites over 3 program waves from April 1, 2007 to June 30, 2011 in Ontario, Canada. Time series analyses of outcomes before and after the program and difference-in-differences analyses comparing changes in program sites with control sites were conducted.

## Results

In before-after models among program sites alone, 90^th^ percentile ED LOS did not change in Wave 1 (-14 minutes [95% CI -47, 20]) but decreased after Wave 2 (-87 [95% CI -108, -66]) and Wave 3 (-33 [95% CI -50, -17]); median ED LOS decreased after Wave 1 (-18 [95% CI -24, -12]), Wave 2 (-23 [95% CI -27, -19]), and Wave 3 (-15 [95% CI -18, -12]); in all Waves, decreases were observed in time to physician assessment, left without being seen rates, and 72-hour ED revisit rates. In the difference-in-difference models, where changes in program sites were compared with controls, the program was associated with no change in the 90^th^ percentile ED LOS in Wave 2 (17 [95% CI -0.2, 33]) and increases in Wave1 (23 [95% CI 0.9, 45] and Wave 3 (31 [95% CI 10, 51]); modest reductions in median ED LOS in Waves 2 and 3 alone; and a decrease in time to physician assessment in Wave 3 alone.

## Conclusions

Although the program reduced ED waiting times, it appeared that its benefits were diminished or disappeared when compared with control sites, which were exposed to system-wide initiatives such as public reporting and pay-for-performance. This study suggests that further evaluation of the effectiveness of Lean methods in the ED is warranted before widespread implementation.

